# H2A.Z and H3:K56Q Affect Transcription Through Chromatin and Yeast FACT-Dependent Nucleosome Unfolding

**DOI:** 10.3390/ijms262210887

**Published:** 2025-11-10

**Authors:** Dmitrii Afonin, Elizaveta R. Ukrainets, Elena Kotova, Nadezhda S. Gerasimova, Grigoriy A. Armeev, Mikhail P. Kirpichnikov, Alexey V. Feofanov, Vasily M. Studitsky

**Affiliations:** 1 Bioengineering Department, Faculty of Biology, Lomonosov Moscow State University, 119234 Moscow, Russia; dmitrii.afonin@fccc.edu (D.A.); shordome@gmail.com (N.S.G.);; 2 Nuclear Dynamics and Cancer Program, Cancer Epigenetics Institute, Fox Chase Cancer Center, Philadelphia, PA 19111, USA; elizasofia@mail.ru (E.R.U.);; 3 Institute of Gene Biology RAS, 34/5 Vavilov Str., 119334 Moscow, Russia; 4 Shemyakin-Ovchinnikov Institute of Bioorganic Chemistry, Russian Academy of Sciences, 117997 Moscow, Russia

**Keywords:** FACT, nucleosome unfolding, +1 nucleosome, H2A.Z, H3K56Ac, transcription initiation, histone turnover

## Abstract

Yeast +1 nucleosomes positioned at transcription start sites must be reorganized to allow transcription initiation. Nucleosome reorganization involves multiple factors including histone chaperone FACT (FAcilitates Chromatin Transcription), histone acetylation, and histone variant H2A.Z; however, the mechanism of this process is not fully understood. Here we investigated nucleosome unfolding in the presence of these factors by combining biochemical assays with single-particle Förster resonance energy transfer (spFRET) microscopy. The presence of the H3:K56Ac mimic (H3:K56Q) alone or together with H2A.Z (but not H2A.Z alone) facilitates the Nhp6-dependent unfolding of nucleosomes by FACT. In contrast to canonical nucleosomes, the unfolding of nucleosomes with the studied variant histones promotes the eviction of core histones from nucleosomal DNA. Furthermore, H2A.Z alone or in synergy with H3:K56Q facilitates transcription through a nucleosome as efficiently as FACT facilitates transcription through canonical nucleosomes. The data suggest that FACT, together with H3:K56 acetylation and H2A.Z, unfold promoter nucleosomes and participate in the eviction of histones to increase the accessibility of the transcription start site, thereby stimulating transcription initiation and possibly early elongation.

## 1. Introduction

The primary level of DNA compaction in the nucleus of most eukaryotes is represented by nucleosomes. An intact nucleosome is so dynamic that it can slide along DNA [1,2,3] and partially or completely disassemble [4]. Despite this dynamicity, nucleosomes prevent the indiscriminate initiation of transcription and replication; therefore, nucleosomes require chromatin reorganization factors which strongly facilitate these genomic processes [5,6,7]. Thus, nucleosomes play an essential role in shielding DNA from unwarranted interactions with transcription machinery; therefore, the assembly of the pre-initiation complex (PIC) and transcription initiation at a transcription start site (TSS) occur within a specific nucleosomal context. Promoters supporting the initiation of transcription by the RNA polymerase II (RNAPII) on the majority of *Saccharomyces cerevisiae* genes contain a region depleted of nucleosomes (Nucleosome-Depleted Region, NDR) due to intrinsic DNA sequence features and ATP-dependent remodeling activities [8,9] and are flanked by strongly positioned +1 and −1 nucleosomes [9]. The yeast TSSs are obstructed by the +1 nucleosome and are localized to approximately one DNA helical turn from the promoter-proximal boundary of the nucleosomal DNA [10]. Hence, the transition from the PIC at NDR to transcription initiation requires the reorganization of the +1 nucleosome [11,12]; however, the mechanism of this process remains to be fully elucidated. The first steps of PIC engaging with the +1 nucleosome are mediated by the translocase activity of the general transcription factor TF_II_H, which detaches two turns of nucleosomal DNA from the nucleosome; but this activity is not sufficient to overcome the nucleosomal barrier and initiate transcription effectively [13].

A variant histone H2A.Z is selectively incorporated into nucleosomes flanking NDR in the direction of transcription [10,14], serving as a hallmark of nucleosomes that encompasses TSS [15]. Moreover, the presence of H2A.Z is crucial for the rapid transcriptional activation of various inducible yeast genes [16,17,18,19]. Histone H2A.Z reduces the barrier presented by the +1 nucleosome to the passage of RNAPII in vivo in Drosophila [11], and according to in silico studies, facilitates the unwinding of nucleosomal DNA [20,21]. In addition, +1 nucleosomes exhibit rapid replication-independent turnover of histone H3, a central component of the nucleosome histone core, suggesting that a complete disassembly of this nucleosome occurs during transcription initiation [22]. The elevated rates of histone turnover at the +1 nucleosome in yeast are concomitantly associated with an ancillary histone modification: the acetylation of histone H3 lysine 56 (H3:K56Ac) [23,24]. This modification represents a distinct post-translational modification inducing alterations on the globular surface of the histone octamer [7] near the entry–exit sites of nucleosomal DNA [25,26]. H3:K56Ac-containing nucleosomes are also characterized by increased dynamics [27] and sensitivity to micrococcal nuclease [28]. The acetylation of H3:K56 has been shown to facilitate the intermittent uncoiling of up to 15 bp from the histone octamer at the nucleosomal edges, a phenomenon described as “nucleosomal breathing” [25,26]. Collectively, these observations indicate an increased instability of nucleosomes containing H2A.Z and H3:K56Ac, establishing a link between H3:K56Ac-mediated histone exchange at the promoter-proximal nucleosome and the efficiency of transcription initiation [29]. Consequently, nucleosomes containing both H2A.Z and H3:K56Ac emerge as pivotal subjects in exploring the mechanisms of +1 nucleosome disassembly during the onset of transcription.

Current in vivo studies also underline a connection between PIC progression and histone turnover at the yeast +1 nucleosome, involving blocking the PIC assembly (by the removal of the TATA-binding protein) or preventing the phosphorylation of the C-terminal domain (CTD) of the RNAPII core subunit (Rpb1) by Kin28 kinase slow histone turnover and causing H2A.Z accumulation at promoters [30,31,32]. Kin28 kinase, a component of the TF_II_H helicase complex, conducts the Ser-5 phosphorylation of Rpb1 CTD heptapeptide repeats which facilitate RNAPII interaction with the histone chaperone FACT (FAcilitate Chromatin Transcription) and is proposed to be involved in the recruitment of FACT to the promoter [33,34]. It has also been demonstrated that in vivo FACT is recruited to +1 nucleosomes that are partially unfolded from the TSS-proximal side [35], suggesting an additional mechanism for FACT targeting to internal nucleosome sites freed from nucleosomal DNA, such as by the helicase activity of the PIC-TH_II_H complex [13].

FACT is a conserved histone chaperone, which is widely present in eukaryotes [36,37,38]. Yeast FACT is a heterodimer of two subunits Spt16 and Pob3. It does not bind to intact nucleosomes [39], but binds to nucleosomes with exposed H2A/H2B dimers in the dyad region. In the last case, FACT encircles the side surfaces of the nucleosome [40], while the C-terminal domain of the Spt16 subunit forms contacts with the DNA-binding surface of the H2A/H2B dimer [41]. Yeast FACT together with the HMG-box protein Nhp6 unfolds nucleosomes containing canonical histones in an ATP-independent, reversible manner [39,42,43]. Nucleosomes following the promoter-proximal +1 nucleosome (+2, +3, etc.) are characterized by a gradual decrease in H2A.Z occupancy [10,30]. According to in vivo studies, H2A.Z-containing nucleosomes from gene bodies are discriminated by elongating RNAPII, acting in complex with FACT and evicting H2A.Z-H2B dimers from nucleosomes [44]. However, the reorganization of H2A.Z-containing nucleosomes by FACT and the dynamics of RNA polymerase transcribing through H2A.Z-containing yeast nucleosomes have not been explored in vitro.

In this work, we perform a comparative study of the unfolding of yeast nucleosomes containing H2A.Z and/or the H3:K56Ac mimic (H3:K56Q) by the yeast histone chaperone FACT and analyze the efficiency of transcription through these nucleosomes to evaluate the possible contribution of these processes to transcription initiation and early elongation by RNAPII through the +1 nucleosome. Our studies revealed that both H2A.Z and H3:K56Ac facilitate nucleosome unfolding and transcription in vitro. Based on the data, we propose a mechanism explaining the consequences of FACT recruitment to the promoter in the context of transcription initiation, RNAPII progression, and histone turnover on the +1 nucleosome.

## 2. Results

### 2.1. The Structure of Yeast Nucleosomes Is Minimally Affected by the Presence of Histones H2A.Z, H3:K56Q, or Their Combination

Previous studies highlighted an instability of yeast nucleosomes relative to their animal counterparts, attributable to specific amino acid variances [45,46,47]. Therefore, yeast nucleosomes, being less stable, are less frequently employed in studies examining interactions with yeast proteins in vitro [46]. In our study, we decided to preserve the yeast origin of all proteins and, accordingly, paid special attention to avoid conditions leading to the artificial destabilization of nucleosomes. In the preliminary single-particle Förster resonance energy transfer (spFRET) microscopy experiments, we found that yeast mononucleosomes became slightly destabilized after 10 min incubation at 30 °C but remained stable at temperatures below 25 °C. Considering this, nucleosomes were incubated with or without yeast FACT (Spt16/Pob3 dimer) and Nhp6 at 25 °C. The unfolding of yeast mononucleosomes by the yeast FACT complex was studied using spFRET microscopy at 21 °C, in contrast to the previous study of the unfolding of nucleosomes containing chicken erythrocyte or *Xenopus laevis* histones that were performed at 30 °C [42,43].

**Figure 1:**
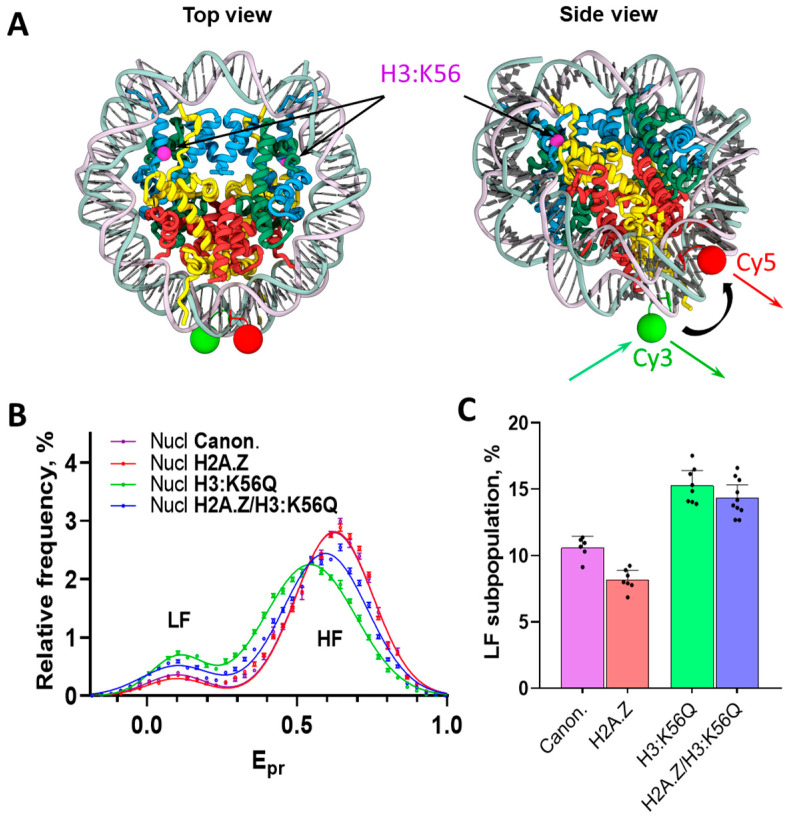
Analysis of nucleosome unfolding by FACT using spFRET microscopy. (**A**) Top and side views of the nucleosome (created using PDB structure 8RGM) containing fluorescent labels Cy3 and Cy5 at positions +35 bp (Cy5, FRET acceptor, red circles) and +112 bp (Cy3, FRET donor, green circles) from the beginning of the nucleosome positioning sequence. The K56 residues of histones H3 are marked using magenta circles. Excitation of Cy3 leads to FRET from Cy3 to Cy5 if the labels are close to each other in the nucleosome (shown by green and red arrows). Detection of particles with high and low proximity ratios E_pr_ is carried out to study the conformation of nucleosomal DNA in single particles using spFRET microscopy. (**B**) Frequency distributions of nucleosomes (E_pr_ profiles) for samples containing canonical core histones (Canon.), H2A.Z, H3:K56Q, or H2A.Z/H3:K56Q measured by spFRET microscopy. Data points are mean ± SEM (*n* = 6–10), data are approximated by two Gaussians. Peaks corresponding to nucleosomes with low (LF) and high (HF) FRET are indicated. (**C**) Quantitation of LF subpopulations in the studied samples (panel B), mean ± SEM (*n* = 6–10). The results of individual measurements are indicated by dots.

For the spFRET microscopy, nucleosomes containing a pair of fluorescent tags localized at positions +35 bp (Cy5) and +112 bp (Cy3) from the beginning of the nucleosome positioning sequence [48] were assembled (Figure 1A, Supplementary Figure S1).

**Figure 2:**
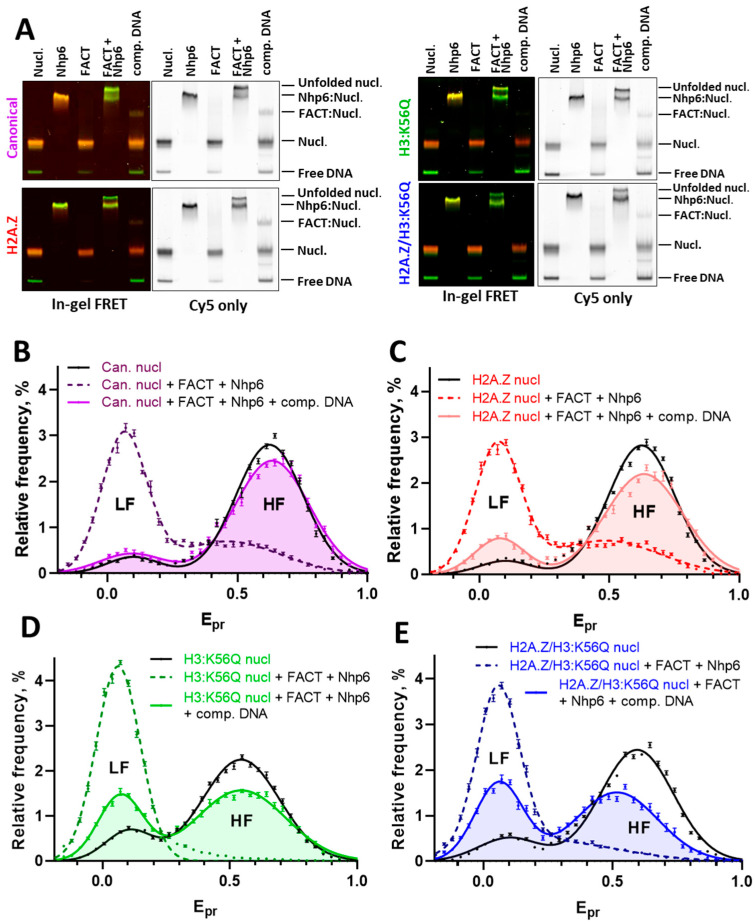
H2A.Z and H3:K56Q partially inhibit refolding of FACT-unfolded nucleosomes. (**A**) Analysis of FACT-dependent unfolding and refolding of the studied nucleosomes (Nucl.) by EMSA. Fluorescently labeled nucleosomes were incubated in the presence of Nhp6, FACT, or Nhp6:FACT. DNA competitor (comp. DNA) was added after nucleosome unfolding by Nhp6:FACT to reverse the unfolding. In-gel FRET: Fluorescence was excited at the 532 nm wavelength and detected in two channels—green (using a 580 nm filter) and red (using a 670 nm filter). Images were merged and qualitatively show FRET for the studied nucleosomes. FRET increases when the color of the band changes in the row green–yellow–orange–red (and vice versa). Cy5 only: fluorescence was excited at 632 nm and detected with a 670 nm filter measuring the emission of Cy5 only. (**B**–**E**) E_pr_ profiles of canonical (Can. nucl) (**A**), H2A.Z-containing (**B**), H3:K56Q-containing, (**C**) and H2A.Z/H3:K56Q-containing (**D**) nucleosomes as well as their complexes with FACT (Spt16/Pob3 dimer) and Nhp6 before and 20 min after addition of competitive DNA. The areas under the E_pr_ profiles of samples Nucl + FACT + Nhp6 + competitor DNA are shaded. Data points are mean ± SEM (*n* = 6–10), data are approximated by two Gaussians. LF and HF indicate peaks corresponding to nucleosomes with low and high FRET, respectively.

The labeled nucleotides were localized far enough from the boundaries of a nucleosome to guarantee that the distances between them reflect rearrangements in the structure of nucleosomes and are not the subject to random fluctuations because of the “breathing” of nucleosomes [42,49,50]. This labeling strategy provides a sensitive probe for detecting large-scale rearrangements occurring within the nucleosome core. Assembled nucleosomes containing either canonical yeast core histones, H2A.Z, or H3:K56Q or H2A.Z/H3:K56Q were characterized using spFRET microscopy (Figure 1B,C). To perform this, proximity ratio (E_pr_) values were calculated for all measured nucleosomes in each experiment and presented as E_pr_ profiles (i.e., relative frequency distributions of nucleosomes by the E_pr_ value). The E_pr_ value is the FRET efficiency without correction for quantum yields of fluorophores and the sensitivity of detectors. As established in prior research [42], intact nucleosomes are characterized by the presence of two peaks in the E_pr_ profiles, corresponding to low (LF) and high (HF) E_pr_ values (Figure 1B). A small subpopulation of particles with a low E_pr_ corresponds to histone-free DNA (Figure 2A); intact nucleosomes are characterized by a high E_pr_ [42]. Although the presence of H3:K56Q alone or in combination with H2A.Z caused a slight increase in the content of free DNA in the corresponding nucleosome samples (probably because of the disruption of DNA-H3 contacts at DNA entry/exit sites and the decrease in nucleosome stability [25]), over 85% of the measured signals in the experimental nucleosomes samples corresponded to the intact nucleosomes (Figure 1C).

**Figure 3:**
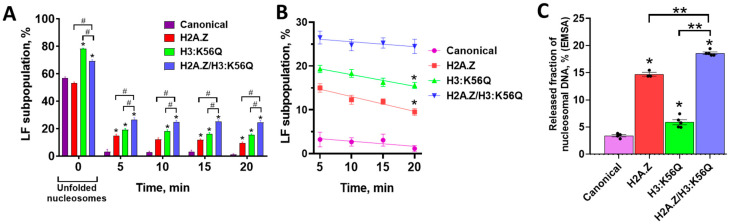
Additive effects of H2A.Z and H3:K56Q on FACT-dependent nucleosome unfolding and refolding. (**A**) LF subpopulations of particles are shown after the reorganization of various nucleosomes by the Nhp6:FACT complex (0 min) and in the course of the reversal of this reorganization at different time points (5–20 min) after the addition of competitor DNA. Incorporation of the variant histone H2A.Z into nucleosomes does not affect nucleosome reorganization but hampers the reversal of this reorganization in the presence of the DNA competitor. The H3:K56Q acetylation mimic and the H2A.Z/H3:K56Q combination facilitate the reorganization of nucleosomes and impede the recovery of their original structure. Background values of LF particles (LF subpopulations shown in Figure 1B) were subtracted from the presented data. Data are mean ± SEM (*n* = 6–10). * *p* < 0.005—significance of difference compared to nucleosomes containing canonical histones; # *p* < 0.005. (**B**) Kinetics of the slow stage of the reduction in subpopulations of reorganized nucleosomes (LF subpopulations) following the addition of competitor DNA to complexes between nucleosomes and Nhp6:FACT. Data are mean ± SEM (*n* = 6–10) taken from panel A; * *p* < 0.005—significance of difference compared to the 5 min point after the addition of competitor DNA. (**C**) Quantitative analysis of nucleosomal DNA released after reversal of unfolding of canonical and H2A.Z/H3:K56Q-containing nucleosomes. The amounts of nucleosomes and histone-free DNA in the gels were quantified using OptiQuant v4.0 after excitation of the acceptor, Cy5, with a red laser (633 nm). Data are mean ± SEM (*n* = 3–6), dots represent individual measurements. * *p* < 0.005—significance of difference compared to nucleosomes containing canonical histones; ** *p* < 0.0001.

Yeast FACT (Spt16/Pob3 dimer) does not interact with yeast nucleosomes on its own, whereas Nhp6 binds nucleosomes, forming a slow-moving fraction of particles in the electrophoretic mobility shift assay (EMSA) (Figure 2A). However, Nhp6 and yeast FACT alone do not affect the structure of nucleosomes near fluorescent labels, as indicated by the lack of changes in the E_pr_ profiles in the presence and absence of Nhp6 or FACT (Supplementary Figure S2A). In contrast, the Nhp6-mediated unwrapping of canonical yeast nucleosomes in the presence of FACT is nearly complete and almost entirely reversible after the addition of the DNA competitor (Figure 2B and Figure 3A). The results agree with the findings which were previously obtained for nucleosomes with histones from chicken erythrocytes and canonical *X. laevis* histones [42,43]. The reversal of yeast nucleosome reorganization is also accompanied by the formation of a small fraction of putative FACT–nucleosome complexes (described in [51]), in which nucleosomes, according to in-gel FRET data, restored their intact structure (Figure 2A). The spFRET analysis revealed a fast refolding of most canonical nucleosomes within less than 5 min (Figure 3A).

### 2.2. The Presence of Variant Histone H2A.Z Hampers Refolding of FACT-Unfolded Nucleosomes

As expected, according to the spFRET analysis, neither FACT, nor Nhp6 alone markedly affect the structure of intact and H2A.Z-containing nucleosomes (Supplementary Figure S2B). The partial reorganization of the nucleosome structure by Nhp6, which is observed in EMSA with in-gel FRET analysis (yellow band, Figure 2A), is likely because of the weaker stability of H2A.Z-containing nucleosomes compared with those containing canonical histones and the more structure-disturbing effect of EMSA compared with spFRET microscopy.

Similarly to the canonical nucleosomes, the reorganization of H2A.Z-containing nucleosomes by the FACT:Nhp6 complex results in the formation of a fraction of unfolded nucleosomes that are slow-moving in gel and have a reorganized structure according to the in-gel FRET analysis (Figure 2A). The comparative spFRET analysis of H2A.Z-containing and canonical nucleosomes revealed the similar efficiency of their unfolding by FACT:Nhp6 (Figure 2B,C and Figure 3A). However, the reversal of the reorganization of H2A.Z-containing nucleosomes is characterized by at least two stages: the fast refolding of many nucleosomes within less than 5 min and the slow refolding of the rest of the nucleosomes during the next 15 min. The fast stage of the refolding of H2A.Z-containing nucleosomes occurs with less efficiency than that of canonical nucleosomes, and a subset of the unfolded H2A.Z-nucleosomes (~10% after correction for an initial base level of LF particles) still fails to revert to their original conformation in the 20 min after the addition of concurrent DNA (Figure 2C and Figure 3A,B).

FACT can both assemble and disassemble canonical nucleosomes (reviewed in [38]). Our observations are consistent with earlier in vitro studies on the ability of FACT to incorporate canonical H2A/H2B and variant H2A.Z/H2B dimers into nucleosomes, which showed that FACT can integrate canonical H2A (as well as another variant H2A.X), but not H2A.Z, into nucleosomes [52]. Our experimental system does not allow interference between H2A- and H2A.Z-containing histone dimers in the nucleosome:FACT:Nhp6 complex. Low concentrations of nucleosomes (0.5 nM) are used during spFRET microscopy, which prevent the reassociation of histones with DNA in case of their eviction from the nucleosome.

In summary, at the level of single nucleosome:FACT:Nhp6 complexes, the reorganization of H2A.Z-containing nucleosomes by FACT is not a fully reversible process and/or the refolding kinetics differs from those of canonical nucleosomes (Figure 2B,C and Figure 3A,B).

### 2.3. The Mimic of Acetylation H3:K56Q Facilitates Nucleosome Unfolding by FACT and Inhibits Nucleosome Refolding

The acetylation of H3:K56, distinctive from promoter nucleosomes, as well as of nucleosomes localized at the 3′ ends of genes [23,24,53], belongs to a group of post-translational modifications of core histones, which affects the dynamic properties of nucleosomes. This acetylation weakens the contacts of histone H3 with nucleosomal DNA at its entry/exit sites, which makes the edges of nucleosomal DNA more flexible and the nucleosome as a whole less stable [25]. It was previously suggested that H3:K56Ac can increase the efficiency of nucleosome reorganization by FACT, but this proposition was not confirmed in vitro [51].

The spFRET analysis shows that Nhp6 and FACT alone do not affect the structure of the H3:K56Q-containing nucleosomes (Supplementary Figure S2C), while in-gel FRET demonstrates a moderate disturbance of the nucleosome structure in the complex with Nhp6 (Figure 2A). Yeast FACT and Nhp6 together reorganize nucleosomes containing the mimic of acetylation H3:K56Q (Figure 2A,D), and, according to spFRET microscopy the unfolding of these nucleosomes occurs with a higher efficiency (78%) than the unfolding of either canonical (57%) or H2A.Z-containing (53%) nucleosomes (Figure 2B–D). Also, the incorporation of H3:K56Q significantly inhibits the reversibility of the unfolding, considerably affecting the fast stage of the refolding process (Figure 2D and Figure 3A,B). In 5 min after the addition of the competitor DNA to the FACT:Nhp6-reorganized nucleosomes, the remaining part of the unfolded H3:K56Q nucleosomes (~19%) is six times higher than that of canonical nucleosomes (~3%) (Figure 3A,B). The rates of the slow stage of nucleosome refolding are similar for canonical and H3:K56Q nucleosomes, resulting in the preservation of a significantly higher subpopulation of reorganized H3:K56Q nucleosomes (~16%) in the 20 min after the initiation of refolding (Figure 3B). Thus, the unfolding of H3:K56Q-bearing nucleosomes by Nhp6:FACT is a process that is not entirely reversible, as was observed in nucleosomes containing the H2A.Z histone variant (Figure 2C,D and Figure 3A,B).

In summary, the introduction of H3:K56Q into nucleosomes facilitates their unfolding by FACT and significantly interferes with the reversibility of FACT-mediated nucleosome unfolding.

### 2.4. H2A.Z and H3:K56Q Cooperatively Affect FACT-Dependent Nucleosome Unfolding and Refolding

Nhp6 and FACT alone minimally affect the structure of H2A.Z/H3:K56Q-containing nucleosomes according to spFRET microscopy (Supplementary Figure S2D); however, the effect of Nhp6 on a nucleosome structure is more evident according to in-gel FRET analysis (Figure 2A). As discussed above, this difference can be due to the more disturbing nature of electrophoresis compared with spFRET microscopy, which is further increased by the higher fragility of the structure of nucleosomes, which contain variant histones. The incorporation of both H2A.Z and H3:K56Q into nucleosomes results in a markedly increased efficiency of their unfolding by the FACT:Nhp6 complex compared with canonical nucleosomes (Figure 2B,E and Figure 3A). And yet, this efficiency is significantly attenuated relative to nucleosomes containing solely H3:K56Q. This phenomenon can be attributable to the moderating influence of H2A.Z on the overall efficiency of nucleosome unfolding (Figure 3A). The data suggest that H2A.Z and H3:K56Q independently affect nucleosome unfolding by FACT, likely using mechanistically different pathways leading to opposite effects when both are present.

Although the presence of H2A.Z hampers the unfolding of H2A.Z/H3:K56Q-containing nucleosomes by the FACT:Nhp6 complex compared with H3:K56Q-containing nucleosomes, a synergy between H2A.Z- and H3:K56Q- mediated effects is observed during nucleosome refolding (Figure 3A,B). The cumulative impact of these histone variants on refolding processes results in the preservation of a significantly increased subpopulation of unfolded nucleosomes both after the fast stage (~26%) and during the slow stage (~20%) of the reversal to the original structure of H2A.Z/H3:K56Q-nucleosomes (Figure 3A,B).

In summary, the introduction of H2A.Z and H3:K56Q into the nucleosome significantly enhances the efficiency of FACT-induced unfolding and negatively affects the reversibility of the unfolding. The effects of H2A.Z and H3:K56Q on nucleosome dynamics are additive, indicating that these histone variants independently affect both processes, likely utilizing distinct mechanisms to modulate the unfolding and refolding of nucleosomes in the presence of Nhp6:FACT.

### 2.5. Histones Are Partially Evicted from Nhp6:FACT-Unfolded H2A.Z/H3:K56Q-Containing Nucleosomes

The observed negative effects of H2A.Z and/or H3:K56Q on the refolding of FACT-unfolded nucleosomes could be explained by the following: (a) the formation of stable unfolded nucleosomes containing all or some core histones, (b) the displacement (eviction) of all core histones from nucleosomal DNA. To discriminate between these possibilities, we analyzed the changes in free nucleosomal DNA after FACT-dependent unfolding and the further reversal of this unfolding by adding competitor DNA for all types of nucleosomes by EMSA (Figure 2A). The quantitation of the nucleosomal DNA released after Nhp6:FACT-dependent nucleosome unfolding and refolding shows that a large fraction of H2A.Z/H3:K56Q-containing nucleosomes (~19%) was converted into histone-free DNA (Figure 3C). Comparison with the size of the fraction of the unfolded H2A.Z/H3:K56Q-containing nucleosomes revealed by spFRET microscopy (~24% after 20 min of the refolding, Figure 3A) suggests that the loss of all core histones can account for the majority of the nucleosomal DNA that remain in the unfolded state after the refolding of nucleosomes. A significant increase in the amount of released DNA was also observed for H2A.Z- and H3:K56Q-containing nucleosomes subjected to Nhp6:FACT unfolding, followed by the reversal of this unfolding (Figure 3C).

In conclusion, the Nhp6:FACT-induced unfolding of H2A.Z- and/or H3:K56Q-containing nucleosomes is accompanied by the displacement (eviction) of all core histones from nucleosomal DNA in a significant proportion of nucleosomes.

### 2.6. H3:K56Q and H2A.Z Additively Facilitate Transcription Through a Nucleosome

**Figure 4:**
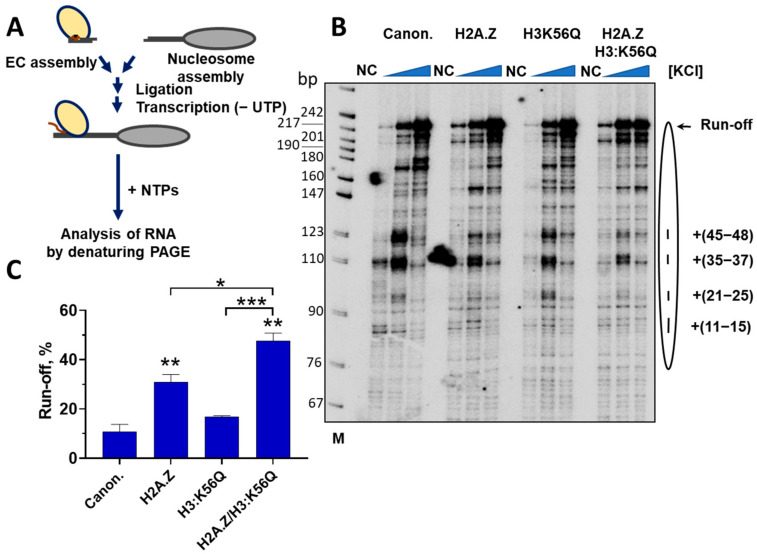
H2A.Z and H3:K56Q cooperate to facilitate transcription through nucleosomes. (**A**) Experimental approach for analysis of transcription through the nucleosome by yeast RNAPII in vitro. RNAPII elongation complexes (ECs) were assembled and ligated to nucleosomes containing H3:K56Q, H2A.Z, H3:K56Q/H2A.Z, or canonical core histones. EC45 complexes were formed in the presence of CTP, ATP, and [α-^32^P]GTP. Transcription was resumed by the addition of an excess of unlabeled NTPs in the presence of various concentrations of KCl, and samples were analyzed by denaturing PAGE. (**B**) Transcription through the nucleosomes containing canonical, H2A.Z, H3:K56Q, and H2A.Z/H3:K56Q histones at different concentrations of KCl (40, 150, and 300 mM). Analysis of pulse-labeled RNA by denaturing PAGE. NC (no chase): analysis of transcripts formed before the chase with unlabeled NTPs. The numbers on the right indicate the positions of the RNAPII active center relative to the promoter-proximal nucleosome boundary in bp. M: pBR322-*Msp*I digest. (**C**) Quantitation of run-off transcripts formed after transcription at 150 mM KCl (mean ± SEM, *n* = 4). ** *p* < 0.005—significance of difference compared to nucleosomes containing canonical histones; *** *p* < 0.0005, * *p* < 0.05.

To study the role of H2A.Z and H3:K56Q during transcription through a nucleosome, a well-established in vitro transcription assay was used [5,54] (Figure 4A). This experimental system faithfully recapitulates many key characteristics of the mechanism of transcription through chromatin observed in vivo [55,56,57].

Nucleosomes containing H3:K56Q, H2A.Z, H3:K56Q/H2A.Z, or canonical core histones were assembled on the DNA template containing the 603-nucleosome positioning sequence that was extensively used for the analysis of the mechanism of transcription through nucleosomes previously [5,54,58,59,60] (Supplementary Figure S4). Yeast RNAPII authentic elongation complexes (ECs) were assembled using synthetic DNA and RNA oligonucleotides and ligated to nucleosomes. RNAPII was advanced to the position of 25 bp upstream of the nucleosome (EC45, the number indicates the length of the RNA product) by incubating these ECs with a limited set of NTPs that lacked UTP and contained [α-^32^P]GTP for the pulsed labeling of RNA. Then the nucleosomal templates were transcribed in the presence of an excess of all unlabeled NTPs in the presence of different concentrations of KCl to evaluate different parts of the nucleosomal barrier to transcription (Figure 4B).

As expected, during the transcription of the nucleosomal templates containing canonical core histones, characteristic pauses were observed at the positions +(11–15), +(21–25), +(35–37), and +(45–48) bp from the promoter-proximal nucleosome boundary (Figure 4B) [54,61]. The transcription of the nucleosomal templates containing the H3:K56Q histone results in a minor change in the pausing pattern and a moderate (~30%) but statistically insignificant increase in the overall efficiency of nucleosome traversal by RNAPII (Figure 4B,C). The transcription of H2A.Z nucleosomes results in a considerable change in the pausing pattern and in a ~3-fold increase in the overall efficiency of nucleosome traversal by RNAPII (Figure 4B,C), suggesting that the presence of H2A.Z strongly affects the dynamic properties of the nucleosome. The presence of both H3:K56Q and H2A.Z in nucleosomes results in a further significant increase in the efficiency of nucleosome traversal by RNAPII (Figure 4B,C), suggesting that these factors affect different aspects of the transcription process. Indeed, H2A.Z preferentially relieves the +(45–48) pausing, while H3:K56Q slightly relieves pausing within the +(35–48) region of nucleosomal DNA (Figure 4B). An increase in the concentration of KCl facilitates the progression of RNAPII through all types of nucleosomes studied, due to the ionic strength-dependent weakening of the contacts between DNA and histones (Figure 4B).

FACT strongly relieves pausing within the +(11–48) region of nucleosomal DNA and efficiently facilitates the progression of RNAPII through nucleosomes containing canonical histones, but does not produce an additional effect on already-facilitated progression of RNAPII through H2A.Z/H3:K56Q-containing nucleosomes (Supplementary Figure S3). This suggests that particular variant histones or their combinations may provide an influence on transcription comparable to that of FACT.

In summary, the data suggests that both H3:K56Q and H2A.Z present in the nucleosomes facilitate RNAPII transcription by relieving the nucleosomal barriers. These factors additively facilitate transcription through the nucleosome: H3:K56Q partially relieves pausing within the +(35–48) region, while H2A.Z preferentially and strongly relieves the +(45–48) pausing.

## 3. Discussion

### 3.1. Overview and Main Findings

H3:K56 acetylation and histone H2A.Z in nucleosomes are predominantly associated with regulatory regions of yeast chromatin, such as +1 nucleosomes at promoters [15]. The results of our study show that the unfolding of yeast nucleosomes containing histone variant H2A.Z and/or the H3:K56 acetylation mimic (H3:K56Q) by histone chaperone FACT and the Nhp6 protein differs considerably from the unfolding of canonical nucleosomes. FACT causes the Nhp6-dependent unfolding of canonical yeast nucleosomes, which is not accompanied by the loss of histones and is fully reversible. In contrast, in the presence of H2A.Z or/and H3:K56Q, the unfolding of nucleosomes by FACT and Nhp6 becomes partially irreversible and is accompanied by the eviction of core histones from a fraction of nucleosomes.

### 3.2. Current Models for +1 Nucleosome Turnover and the Role of FACT

Three non-exclusive models have been put forward to explain the histone turnover at the +1 nucleosome. First, the acetylation of H3:K56 by the Rtt109–Asf1 complex tags newly deposited H3/H4 dimers [24,28,62,63,64,65,66,67,68]. Once an H3:K56Ac dimer enters the +1 nucleosome, it loosens DNA entry–exit contacts, promotes the loss of H3, and sets up a positive-feedback loop in which the vacant site is refilled by another H3:K56Ac dimer [23,24,25]. Because H2A.Z-rich nucleosomes recruit Asf1 [69], the two marks reinforce each other and collectively prime the promoter for fast transcriptional activation. Second, H3:K56Ac alters the behavior of H2A.Z loader SWR1C. Under normal conditions SWR1C swaps H2A/H2B for H2A.Z/H2B stepwise, but H3:K56Ac relaxes this substrate preference, allowing the remodeler to catalyze both forward and reverse exchange [70,71,72]. This bidirectional activity shuttles H2A and H2A.Z dimers in and out of the +1 nucleosome, speeding up turnover. Third, the related remodeler INO80C can remove H2A.Z and reinstall H2A; H3:K56Ac enhances this eviction in vitro [73], although genome-wide studies disagree on how often INO80C carries out the swap in vivo [30,44]. Together, intrinsic destabilization by H3K56Ac, SWR1C-driven bidirectional exchange, and INO80C-mediated H2A.Z removal provide routes that keep the +1 nucleosome dynamic and ready for transcription. However, histone exchange at the +1 nucleosome is linked to transcription initiation according to in vivo studies [30,31,32], and the abovementioned models do not mechanistically explain such connection.

We suggest that one of the factors that couples transcription initiation and histone exchange on +1 nucleosomes in yeast might be the histone chaperone FACT, facilitating transcription initiation due to its Nhp6-dependent ability to irreversibly unfold H2A.Z and H3K56Ac-containing nucleosomes.

### 3.3. FACT-Mediated H2A.Z Destabilization During Elongation of Transcription

In vitro studies demonstrate that FACT selectively removes H2A.Z/H2B dimers from nucleosomes while incorporating canonical H2A, consistent with the participation of FACT in the displacement of H2A.Z during transcription elongation [44,52]. The lower efficiency of the recovery of H2A.Z/H3:K56Q-containing nucleosomes after the reversal of FACT-dependent nucleosome unfolding (Figure 2 and Figure 3) explains the removal of H2A.Z by the FACT complex at gene bodies during transcription elongation in vivo [35,44,74]. Indeed, since H2A.Z/H3:K56Q-containing nucleosomes are apparently less stable than canonical nucleosomes after FACT-dependent nucleosome unfolding, they would be preferentially lost during transcription in the presence of FACT.

### 3.4. Mechanistic Role of Nhp6 and Entry/Exit DNA Contacts

In yeast, the destabilization of nucleosomes required for FACT binding is not mediated solely by elongating RNAPII. Nhp6, a small protein possessing an HMGB1-like DNA-binding domain, collaborates with FACT for large-scale, transcription- and ATP-independent nucleosome remodeling in vitro [39,42,75]. According to a recent model of yeast FACT action, Nhp6 destabilizes the edges of nucleosomal DNA [42] and facilitates the access of FACT to the nucleosome [38]. Our current experiments with the H3:K56Ac mimic with disrupted DNA-H3:K56 contacts (Figure 2D) further supports this hypothesis [42,51] suggesting that the disruption of DNA entry/exit site contacts in nucleosomes promotes Nhp6-dependent nucleosome unfolding by the yFACT complex.

### 3.5. Initiation: Dislodging the +1 Nucleosome Before RNAPolII Transit

The initial phase of transcription in yeast commences with the formation of PIC at NDR [76]. The initiation of transcription is accompanied by the extensive exchange of all core histones [22], likely due to the dissociation of the obstructive +1 nucleosome, which is present at TSS. Given that the FACT complex, aided by Nhp6, can execute nucleosome reorganization autonomously from the elongation activities of RNAPII, it is plausible to postulate its involvement in the dislodgement of +1 nucleosomes before transcription initiation [38]. This conjecture garners indirect support from observations indicating that the interaction of FACT with nucleosomes during transcription elongation leads to the dissociation of the H2A.Z/H2B dimer, a hallmark of the +1 nucleosome [44,52,74]. In temperature-sensitive yeast Spt16 mutants, an accumulation of histones at the +1 nucleosome was noted, suggesting the FACT-dependent removal of TSS-blocking nucleosomes in vivo [77]. This approach also revealed the role of FACT in the activation of *SCB* and *PHO4* promoters: the inhibition of FACT resulted in the delayed eviction of promoter nucleosomes, though it had a negligible impact on the steady-state mRNA levels of genes controlled by these promoters [78,79]. Nonetheless, studies on H3:K56 hyperacetylation indicate that the effects of Lys56 mutations on transcription initiation might be elusive when analyzed via mRNA sequencing but are distinctly observable using nascent transcript sequencing (NET-seq) [29]. A similar situation was noted in research involving yeast mutants H3:K56Q and Pob3:Q308K, which affect the efficacy of nucleosome remodeling by the histone chaperone FACT in vitro; these mutants exhibited pronounced phenotypic effects that were challenging to detect by mRNA-seq [51].

### 3.6. Early Elongation Through +2 and +3 Nucleosomes: Effects and Constraints

In addition to affecting transcription initiation, the presence of some amount of H2A.Z/H3:K56Ac within the +2 and +3 nucleosomes could affect the efficiency of early transcription elongation. Indeed, H2A.Z/H3:K56Q-containing nucleosomes are more efficiently transcribed than canonical nucleosomes (Figure 4B,C). RNAPII encounters transcriptional pausing at the +15 and +45 regions within the nucleosomal DNA, corresponding to regions of strong interactions between histones and DNA [80,81]. FACT significantly eases RNAPII transit across these impediments in vitro, fostering the accumulation of full-length transcription products. RNAPII entry induces partial nucleosome destabilization, which facilitates the binding of the H2A/H2B dimer by the FACT complex, creating an approximate 30 bp window for this interaction [82]. FACT interacts with the nucleosome at the nucleosome dyad region, enveloping the lateral nucleosomal surfaces through interactions between the middle domains (MDs) of its subunits [40]. Additionally, the C-terminal domain (CTD) of the Spt16 subunit forms contacts with the DNA-binding surface of the H2A/H2B dimer [41]. This interaction facilitates DNA bending away from the nucleosome, enabling RNAPII to surmount the +15 pausing site without disassociating the H2A/H2B dimer from the complex [82]. Thus, the primary condition for the binding of FACT to transcribed nucleosomes is the polymerase-induced partial destabilization of the nucleosome [38].

However, the presence of H2A.Z/H3:K56Ac is unlikely to strongly facilitate transcription through the +2 and +3 nucleosomes in vivo because (a) these histones are present only on a fraction of +2 and +3 nucleosomes [30,83], (b) both canonical and H2A.Z/H3K56Ac-containing nucleosome are efficiently transcribed in the presence of FACT (Supplementary Figure S3) that is present on the transcribed genes [77,84].

### 3.7. Integrated Model and Implications for Promoter Opening

**Figure 5:**
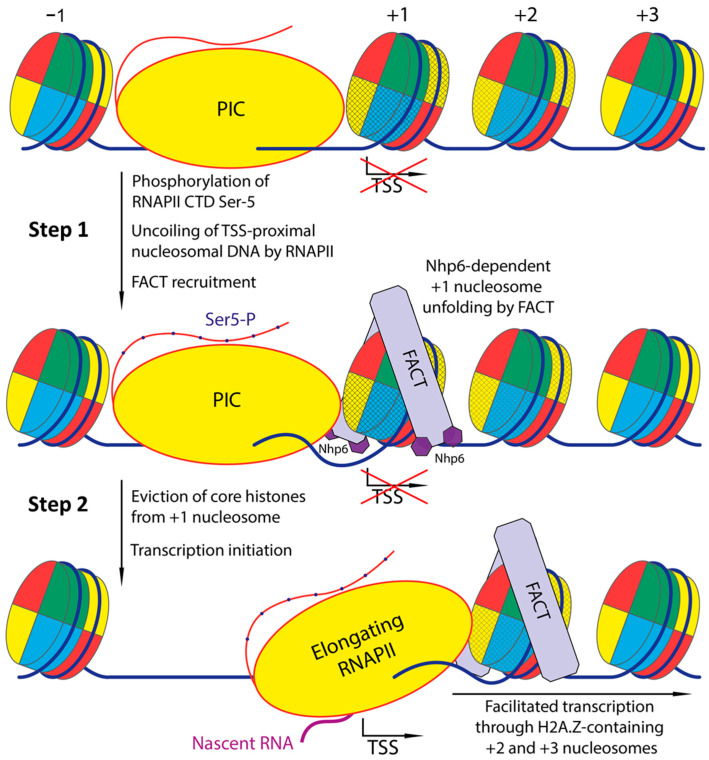
Proposed mechanism of histone turnover and eviction on yeast promoter: histone chaperone FACT in transcription initiation. Step 1: The assembly of PIC is completed after phosphorylation of Ser5 CTD of RNAPII, which likely facilitates recruitment of FACT. The histone chaperone FACT, cooperatively with the site-nonspecific transcription factor Nhp6, irreversibly and ATP-independently reorganizes H2A.Z- and H3K56Ac-containing +1 nucleosomes, most likely with help of other protein factors (see Section 3) releasing nucleosomal DNA and TSS for transcription initiation. Step 2: FACT facilitates transcription through a +2 nucleosome that can also contain the variant H2A.Z histone. H2A.Z and H3:K56Ac histones of +1 and subsequent nucleosomes are indicated by yellow and cyan shading, respectively.

The results of this study suggest a model wherein H3:K56 acetylation (H3:K56Ac) augments Nhp6-mediated nucleosome remodeling by the FACT complex and, in conjunction with H2A.Z, facilitates the removal of the yeast histone octamer, thereby releasing nucleosomal DNA (Figure 5). This model is corroborated by studies demonstrating the PIC-dependent nature of histone turnover at the +1 nucleosome [30,31,32] and the recruitment of FACT to the +1 nucleosome during transcription initiation (via the phosphorylation of Ser-5 RNAPII CTD or nucleosome unfolding by the translocase activity of TFIIH) [13,33,34,35]. This model is consistent with the observed intense turnover of all histones at the +1 nucleosome in vivo [22] and suggests that FACT could facilitate transcription initiation by enhancing the accessibility of TSS within nucleosomal DNA.

## 4. Materials and Methods

### 4.1. Proteins

Octamers containing canonical *S. cerevisiae* histones as well as variant histone H2A.Z and the acetylation mimic H3:K56Q, yeast site-nonspecific transcription factor Nhp6, and yeast FACT (Spt16/Pob3 dimer), obtained as described earlier [85,86,87], were kindly provided by Dr. T. Formosa (University of Utah). Yeast RNAPII was purified as described previously [88].

### 4.2. Nucleosome Assembly for spFRET Microscopy and Gel Purification

Nucleosomes were assembled on 147 bp DNA fragments containing the variant of nucleosome positioning sequence Widom 603 (603-42A) [49,89]. The nucleosomal DNA was obtained using PCR under conditions specific for amplification of short fragments [90] and purified with the Cleanup Standard kit (Evrogen, Moscow, Russia). Fluorescent Cy5 and Cy3 tags were incorporated at the positions +35 and +112 of nucleosomal DNA using the primers previously used in [48]. To assemble nucleosomes, octamers of recombinant histones were mixed with 147 b.p. DNA at molar ratio 1.5:1 and subjected to stepwise dialysis with decreasing salt concentrations [54]. Then nucleosomes were separated in 5% PAGE at 4 °C in HE buffer: 10 mM HEPES-Na (pH 8.0, adjusted by HCl), 0.2 mM EDTA. The position of nucleosomes in the gel was determined by the Typhoon Trio fluorescence imager (GE Healthcare, Chicago, IL, USA). The gel segment containing nucleosomes was excised, and the nucleosomes were extracted into HE buffer containing 100 mg/mL of bovine serum albumin [42].

### 4.3. Nucleosome Unfolding by FACT, spFRET Microscopy, and Analysis of Electrophoretic Mobility of the Complexes

Nucleosomes were incubated (25 °C, 10 min) with 130 nM FACT (Spt16/Pob3 dimers) and 1.3 μM Nhp6 in a buffer 20 mM HEPES-Na (pH 8.0, adjusted by HCl), 100 mM KCl, 100 μM TCEP-HCl, 200 mg/mL BSA, and 10% *w*/*v* sucrose. The nucleosome concentration was 0.5 nM in the spFRET experiments. Binding of Nhp6:FACT to nucleosomes was reversed by adding the competitor, salmon sperm DNA (up to 675 ng/μL), to the reaction mixture. spFRET microscopy and primary analysis of the data were performed as described [91].

Each experiment was carried out with at least 6 replicates. A set of E_pr_ values for analyzed particles (2000–4000 particles per replicate) in each replicate was graphically presented as the relative frequency distribution of nucleosomes by the E_pr_ value (E_pr_ profile). The E_PR_ profiles were averaged (mean ± SEM, *n* > 6) and presented in Figures. Each E_pr_ profile was fitted with a superposition of two Gaussian curves, corresponding to particles with LF (E_pr_ peak ~ 0.1) and HF (E_pr_ peak ~ 0.65). The content of the subpopulation of LF particles was determined by calculating the ratio of the area under the corresponding Gaussian peak to the area under the entire E_pr_ profile (in percentage). Values of the subpopulations of LF particles were averaged (mean ± SEM, *n* > 6). To determine the significance of the revealed differences in the subpopulations of LF particles, an unpaired two-tailed *t*-test was used. The difference between the values was considered as significant if *p* < 0.05. For statistical calculations and presentation of results, GraphPad Prism 8 v8.0.1 was used. Both control and experimental measurements for each nucleosome type were performed on the same nucleosome sample for one day.

Electrophoretic mobility shift assay (EMSA) experiments were performed in the same buffer and with the same concentrations of proteins as the spFRET microscopy experiments, except for the concentration of fluorescently labeled nucleosomes, which was 2 nM. The difference in nucleosome concentrations was dictated by the difference in the sensitivity of the equipment used to detect fluorescence. Samples containing (1) 2 nM nucleosomes, (2) 2 nM nucleosomes with 1.3 µM Nhp6, (3) 2 nM nucleosomes with 130 nM Spt16/Pob3, or (4) 2 nM nucleosomes with 130 nM Spt16/Pob3 and 1.3 μM Nhp6 were incubated for 10 min in buffer at 25 °C, then transferred to ice and applied to the gel. In sample (5), which was prepared to assess the reversibility of nucleosome unfolding, 2 nM nucleosomes were mixed with 130 nM Spt16/Pob3 and 1.3 μM Nhp6 in the same buffer, incubated for 10 min, and competitive DNA was added (up to 675 ng/μL). Then the sample was incubated for an additional 10 min, transferred to ice, and applied to the gel. EMSA of nucleosome complexes with FACT was performed at 4 °C in 4% PAGE containing 10% glycerol in HE buffer. Visualization and analysis of EMSA results were carried out with the software ImageJ v1.52p and OptiQuant v4.0. The fraction of DNA molecules released from nucleosomes after reversal of unfolding, *∂*, was calculated using the formula:

(1) $$\partial =\frac{{DNA}_{2}-{DNA}_{1}}{{Nucl}_{1}}$$

where *Nucl*_1_ is the fraction of nucleosomes in the control sample (measured during excitation and emission of only the Cy5 label), *DNA*_1_ and *DNA*_2_ are the fractions of free DNA in control samples and in samples after reversal of unfolding, respectively. Experiments were repeated at least three times, and the calculated values of *∂* were averaged (mean ± SEM, *n* = 3–6). To determine the significance of the differences in *∂* for the studied nucleosomes, a one-way ANOVA with Tukey’s multiple comparisons test was used. The difference between the values was considered as significant if *p* < 0.05.

### 4.4. Transcription of Nucleosomes

DNA templates for assembly of nucleosomes used in the transcription assay were prepared by PCR using plasmid pGEM-3Z/603 containing Widom 603 sequence as a template [89,92]. The resulting sequence of the amplified DNA fragment is presented in Supplemental Figure 4. The fragment was purified in gel and digested with TspRI restriction enzyme (New England Biolabs GmbH, Frankfurt am Main, Germany) to produce the 9 nt 3′-overhang for further ligation of the pre-assembled elongation complexes (ECs) containing yeast RNAPII [58]. Nucleosomes were assembled as described earlier using stepwise dialysis against buffers containing a decreasing concentration of NaCl [93]. Yeast RNAPII was purified as described [94]. The ECs with 9-meric RNA (EC9) were assembled as described earlier using yeast RNAPII [54,95]. Shortly, a template DNA strand with annealed 9-meric RNA and a non-template DNA strand (oligonucleotide sequences are listed in Supplementary Figure S4) were incubated with RNAPII in the transcription buffer (TB; 20 mM Tris HCl, pH 7.9, 5 mM MgCl_2_, 40 mM KCl, and 1 mM β-mercaptoethanol). The EC9 was purified on Ni-NTA agarose beads (Qiagen GmbH, Hilden, Germany) and ligated to the nucleosomes [54,94,95]. RNA was elongated up to 45 nucleotides in the presence of [α-^32^P]GTP (3.000 Ci/mmol, 10 mCi/mL, 3.3uM, Perkin Elmer, Shelton, CT, USA, BLU006H), ATP (10 μM), CTP (2 μM), and GTP (10 μM)-forming EC45 with pulse-labeled RNA. Transcription was resumed by the addition of a large excess of unlabeled NTPs (500 μM) in the presence of 40, 150, or 300 mM KCl and carried out for 10 min. In experiments with yFACT, the protein (0.2 or 0.4 µM) was added to the reaction simultaneously with unlabeled NTPs. Nucleic acids were purified by phenol/chloroform extraction and precipitated in 70% ethanol with the addition of glycogen. Samples were dissolved in a denaturing loading buffer (95% formaldehyde, 0,1% SDS), incubated for 5 min at 95 °C, and analyzed by denaturing PAGE. Pulse-labeled RNA fragments were visualized using a PhosphorImager (BioRad, Hercules, CA, USA). Results were analyzed with the OptiQuant-GelPro program. RNAPII run-offs were calculated as a percentage of total RNAPII pausing during transcription through the nucleosome. The transcription experiments were repeated four times. Relative amounts of run-off RNA were averaged (mean ± SEM, *n* = 4). The significance of the differences in run-off RNA was determined using a one-way ANOVA with Tukey’s multiple comparisons test; differences were considered as significant if *p* < 0.05.

## 5. Conclusions

Our biochemical and single-molecule analyses show that FACT, aided by Nhp6, induces the large-scale, ATP-independent unfolding of yeast nucleosomes. The histone variant H2A.Z and the acetylation mimic H3:K56Q both potentiate this remodeling by reducing its reversibility: a substantial fraction of remodeled particles fails to refold and can lose core histones. In transcription assays, H2A.Z and H2A.Z together with H3:K56Q cooperatively facilitate RNAPII passage by relieving distinct components of the nucleosomal barrier, whereas saturating FACT broadly alleviates pausing. Together, these findings suggest a mechanistic link between promoter-proximal chromatin marks, FACT, and histone turnover at the +1 nucleosome: the recruitment of FACT during early initiation would expose TSS DNA, promote the eviction/turnover of octamers enriched with H2A.Z and H3K56Ac, and thereby stimulate initiation and early elongation. This model integrates histone exchange with PIC progression and provides a framework for understanding how promoter chromatin is primed for rapid transcriptional activation by H2A.Z and acetylation.

## Data Availability

The original contributions presented in this study are included in the article/Supplementary Material. Further inquiries can be directed to the corresponding author.

## Supplementary Materials

The following supporting information can be downloaded at: https://www.mdpi.com/article/10.3390/ijms262210887/s1.

## Abbreviations

The following abbreviations are used in this manuscript:

CTD	C-terminal domain (of Rpb1)
EC	Elongation complex
EMSA	Electrophoretic mobility shift assay
FACT	Yeast FAcilitates Chromatin Transcription complex
H3:K56Ac	Histone H3 with acetylated K56
H3:K56Q	Histone H3 with acetyl mimic substitution K56Q
NDR	Nucleosome-depleted region
PIC	Pre-initiation complex
RNAPII	RNA polymerase II
spFRET	Single-particle Förster resonance energy transfer
TF_II_H	General transcription factor IIH
TSS	Transcription start site
