# Supplementary material for: H2A.Z and H3:K56Q Affect Transcription Through Chromatin and Yeast FACT-Dependent Nucleosome Unfolding
Source: Int J Mol Sci. 2025 Nov 10;26(22):10887. doi: 10.3390/ijms262210887 (PMC12652531; doi:10.3390/ijms262210887)
Supplement: Supplementary file 1 [file ijms-26-10887-s001.zip › ijms-3908964-supplementary.pdf]

Title: **H2A.Z and H3:K56Q Affect Transcription through Chromatin and yeast FACT-dependent Nucleosome Unfolding**

Dmitrii Afonin, Elizaveta R. Ukrainets, Elena Kotova, Nadezhda S. Gerasimova, Grigoriy A. Armeev, Mikhail P. Kirpichnikov, Alexey V. Feofanov and Vasily M. Studitsky

SUPPLEMENTARY MATERIALS

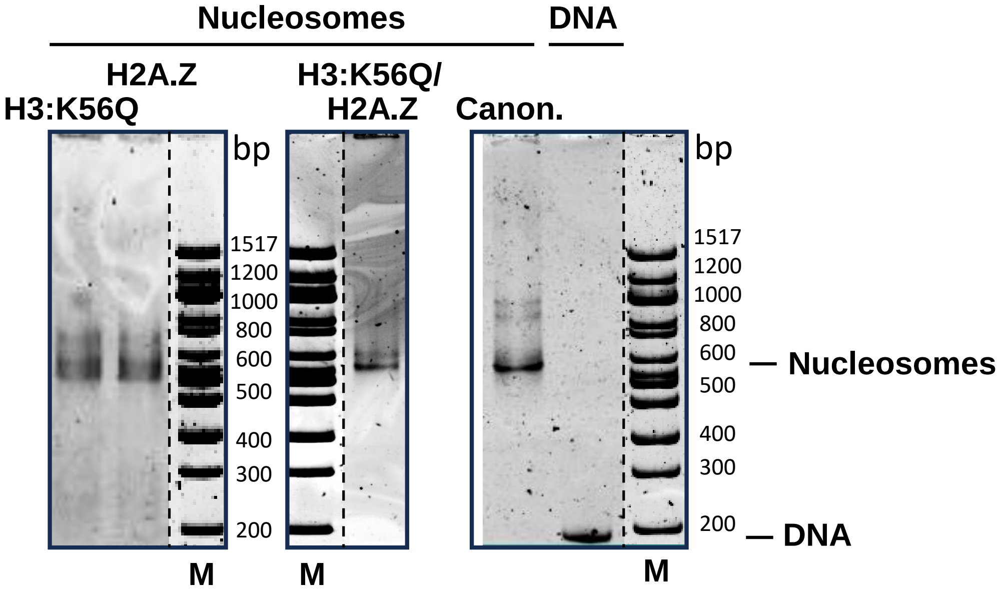

Figure S1. **Characterization of nucleosomal templates for transcription by RNAPII.** Nucleosomes with H3K56Q, H2A.Z**,** H3K56Q/H2A.Z or canonical histones were reconstituted on end-labeled 603 DNA templates and analyzed by native PAGE. Positions of nucleosomes and histone-free DNA in the gel are indicated. M: M: 100- bp DNA ladder (NEB)

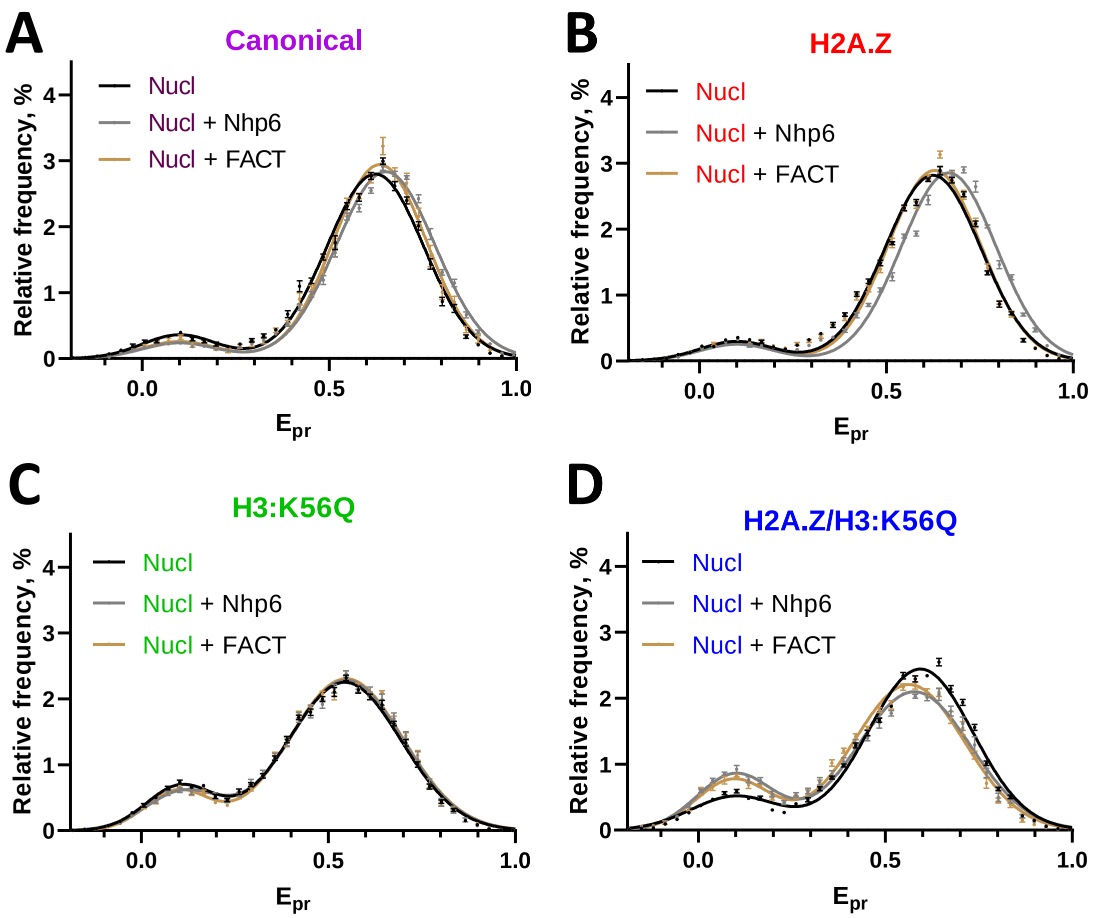

Figure S2. **Nhp6 and FACT alone do not significantly affect nucleosome structure.** E_{PR} profiles of nucleosomes containing canonical yeast core histones (**A**), H2AZ (**B**), H3-K56Q (**C**) or H2AZ/H3:K56Q (**D**) incubated in the presence or in the absence of FACT or Nhp6 protein. Other designations as in **Figure 2A**. Data points are mean±SEM (n=6-10), data are approximated by two Gaussians.

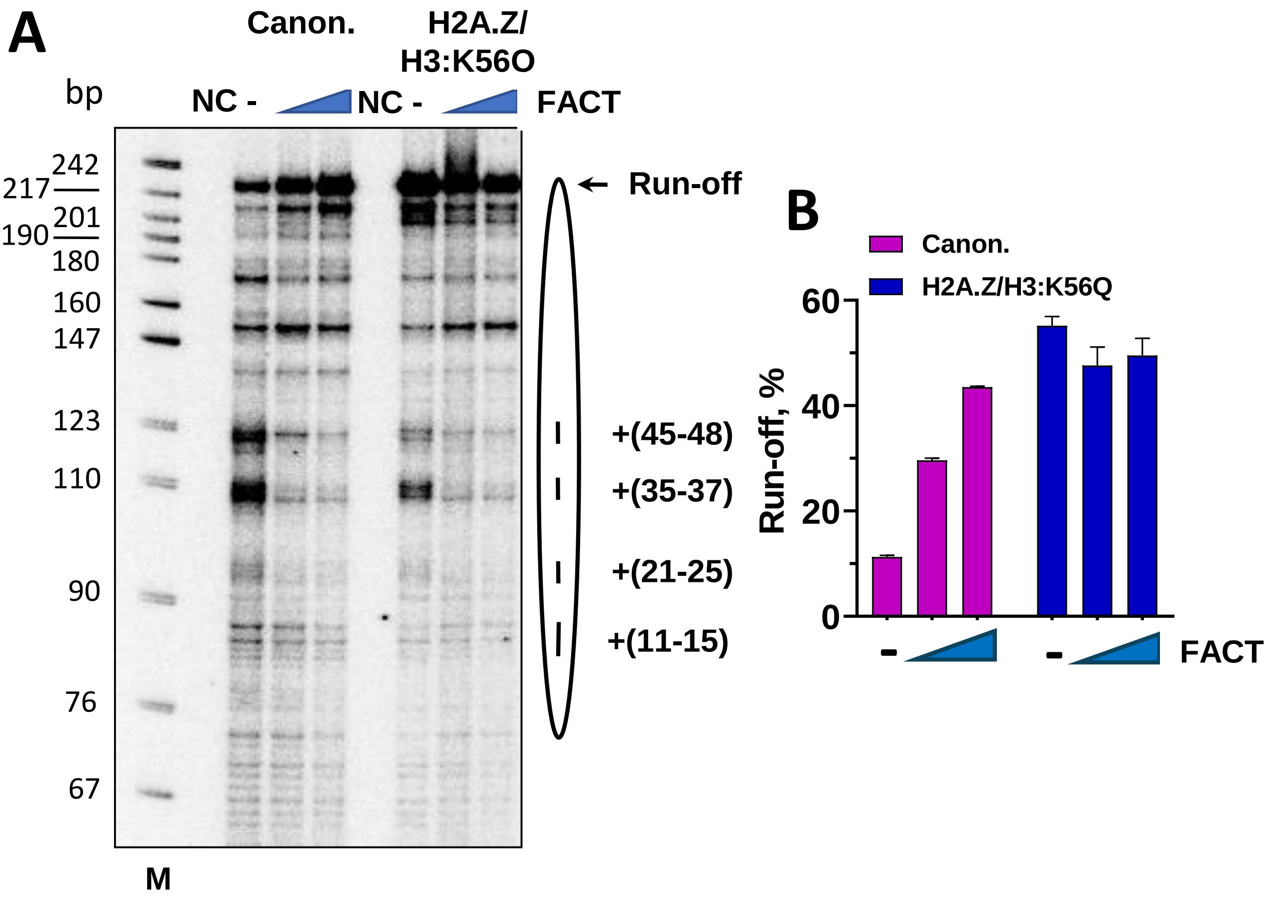

Figure S3. **Transcription through canonical and H2A.Z/H3:K56Q-containing nucleosomes in the presence of FACT.** (**A**) Canonical (Canon.) and H2A.Z/H3:K56Q nucleosomes were transcribed in the absence and in the presence of FACT (0.2 or 0.4 μM) as described in **Figure 4A**. M: pBR322-*Msp*I digest. (**B**) Quantitation of run-off transcripts formed after transcription through canonical and H2A.Z/H3:K56Q-containing nucleosomes in the presence of FACT (0.2 or 0.4 μM).

1. Nucleotide sequence of the DNA fragment for the assembly of nucleosomes:
   5’-CCAGCGTGGG**CACTG**GGATAATCGACACTCTCGGGTG
   CCCAGTTCGCGCGCCCACCTACCGTGTGAAGTCGTCACTCGGGCTTCTAAGTAC
   GCTTAGCGCACGGTAGAGCGCAATCCAAGGCTAACCACCGTGCATCGATGTTGA
   AAGAGGCCCTCCGTCCTTATTACTTCAAGTCCCTGGGGT-3’
   Widom 603 sequence is underlined
   TspRI site is shown in bold

2. Oligonucleotides used for EC9 assembly:
   A. Template DNA strand: 5’-GGTGTCGCTTGGGTTGGCTTTTCGCCGTGTCCCTCTCGATGGCTGTAAGT-3’
   B. 9-meric RNA: 5’-AUCGAGAGG-3’
   C. Non-template DNA strand: 5’-ACTTACAGCCATCGAGAGGGACACGGCGAAAAGCCAACCCAAGCGACACCGGCACTGGG-3’

Figure S4. Nucleic acid components used for *in vitro* transcription assay.
